# DiseaseLinc: Disease Enrichment Analysis of Sets of Differentially Expressed LincRNAs

**DOI:** 10.3390/cells10040751

**Published:** 2021-03-29

**Authors:** Piyush More, Sweta Talyan, Jean-Fred Fontaine, Enrique M. Muro, Miguel A. Andrade-Navarro

**Affiliations:** 1 University Medical Center, Department of Pharmacology, Johannes Gutenberg University Mainz, 55131 Mainz, Germany; piyusmor@uni-mainz.de; 2 Faculty of Biology, Johannes Gutenberg University of Mainz, 55128 Mainz, Germany; fontaine@uni-mainz.de (J.-F.F.); muro@uni-mainz.de (E.M.M.); 3 Bioinformatics Core Unit (BCU), Max Planck Institute for Heart and Lung Research, 61231 Bad Nauheim, Germany; Sweta.Talyan@mpi-bn.mpg.de

**Keywords:** lincRNAs, diseases, enrichment analysis, web tool

## Abstract

Long intergenic non-coding RNAs (LincRNAs) are long RNAs that do not encode proteins. Functional evidence is lacking for most of them. Their biogenesis is not well-known, but it is thought that many lincRNAs originate from genomic duplication of coding material, resulting in pseudogenes, gene copies that lose their original function and can accumulate mutations. While most pseudogenes eventually stop producing a transcript and become erased by mutations, many of these pseudogene-based lincRNAs keep similarity to the parental gene from which they originated, possibly for functional reasons. For example, they can act as decoys for miRNAs targeting the parental gene. Enrichment analysis of function is a powerful tool to discover the functional effects of a treatment producing differential expression of transcripts. However, in the case of lincRNAs, since their function is not easy to define experimentally, such a tool is lacking. To address this problem, we have developed an enrichment analysis tool that focuses on lincRNAs exploiting their functional association, using as a proxy function that of the parental genes and has a focus on human diseases.

## 1. Introduction

Long intergenic non-coding RNAs (lincRNAs) are transcripts longer than 200 nucleotides, whose function is generally not related to their translation into proteins. They have been found to be involved in a variety of cellular processes, including the regulation of gene expression and splicing [1], and, naturally, mutation and malfunctioning of lincRNAs can result in diseases like cancer [2]. For this reason, the study of lincRNA levels in diseases using high-throughput sequencing technologies has become part of the investigation of mechanisms of disease, and lincRNAs are sought as disease markers and therapeutic targets in tissues [3] or circulating [4].

Omics data is very adequate for the study of the mechanisms and effects of disease and mutation due to the breadth of molecular elements from which information is obtained, for example, altered levels of expression of thousands of human transcripts or proteins. Relating differentially expressed molecules with public data on their functions and associations with disease allows the finding of potential markers for disease. Given the usually large amount of data points and functional information available in the databases, computational approaches have been developed to exhaustively explore the data. One of the most widely used methods is enrichment analysis, which is typically applied to evaluate the statistical significance of functions associated with transcripts or proteins in sets of differentially expressed genes or proteins, respectively [5,6].

The application of enrichment analysis to lincRNAs seems to be a desirable goal, but this has been hampered by their lower level of functional annotation compared to coding RNAs and proteins, which is due to their less specific expression and mode of action. As a result, enrichment analysis tools for lincRNAs are currently lacking.

However, it is possible to associate lincRNAs with potential target genes, thus, with the function of their target genes, taking advantage of the way lincRNAs arise in evolution. There is evidence that many lincRNAs have originated from decaying pseudogenes [7], and their conservation with respect to the parental gene reveals patterns that are consistent with their activity as RNA competitors of the parental transcript [8,9]. Several mechanisms are possible for this competitive effect with different regulatory outcomes: pseudogene expression of antisense transcripts or siRNAs may reduce translation of the parental gene’s sense transcript; and sense pseudogene transcripts sharing miRNA target sites with the parental sense transcript may compete for miRNAs targeting the parental gene’s transcript, allowing an increase in its translation [10]. 

Due to such functionally-relevant conserved sequence similarity between pseudogenes and their parental genes, it was possible to devise strategies to identify lincRNAs associated with parental genes based on the comparison of DNA sequences using mutation matrices that model neutral evolution [11]. These associations expand the potential functional annotations of lincRNAs. 

Here, we exploit these expanded associations with a method and server, DiseaseLinc, which takes as an input a list of differentially expressed human transcripts to perform enrichment analysis specific to the lincRNAs identified in the dataset and focused on disease.

## 2. Materials and Methods

A list of associations of lincRNAs to parental protein-coding genes was obtained from previous work [11]. Here, we summarize the methodology used to derive these associations; full details are described in the publication cited.

In previous work, lincRNAs with remnants from protein-coding genes were detected by aligning their translated amino acid sequences (all three open reading frames in the direction of transcription) against the amino acid sequence of proteins. The customized alignment method used a substitution matrix to score sequence divergence events. The matrix was optimized for aligning protein-coding genes with non-coding gene sequences, assuming neutral evolution [12]. As a result, a total of 203 human lincRNA genes corresponding to 164 protein-coding genes and not overlapping any gene were identified and selected. These lincRNAs displayed a significant correlation of expression with their parental protein-coding genes.

### 2.1. Data Sources

UniProtKB IDs of the 164 proteins associated with the list of 203 selected lincRNAs were mapped to Entrez gene IDs. PubMed records associated with these Entrez gene IDs were extracted from gene2pubmed (NCBI FTP site). To extract PubMed records associated with diseases, we used manual annotations of the PubMed records using the medical subject headings thesaurus MeSH. Disease terms were extracted from the branch “C” of the MeSH, and PubMed records annotated with disease MeSH Unique IDs were identified. All downstream calculations were limited to PubMed records associated with at least one lincRNA parental gene and one disease.

### 2.2. Web Server Implementation

All of the data were locally downloaded and stored in an indexed MySQL database. To identify significant associations between lincRNAs and diseases, a one-tailed Fisher’s exact test was performed as described before [13]. The resulting *p*-value was corrected for multiple tests by calculating the false discovery rate (FDR) by the Benjamini and Hochberg method [14]. All statistical analyses were performed in R statistical environment. A web app was created using shiny and shinyjs packages in R.

### 2.3. A Case Study Using Breast Cancer LincRNAs

To demonstrate the clinical relevance of the DiseaseLinc tool, we extracted 72 lincRNAs associated with breast cancer. To correlate their expression with clinical outcome, we obtained a breast cancer lincRNA expression dataset of 79 patients from the atlas of long non-coding RNA in cancer (TANRIC) [15]. FPKM-normalized data with clinical information were obtained. For survival analysis, the expression data were divided into low and high expression groups using a 50% quantile. Kaplan–Meier curves were generated using the R statistical environment [16].

## 3. Results

### 3.1. Web Server Usage

**Figure 1:**
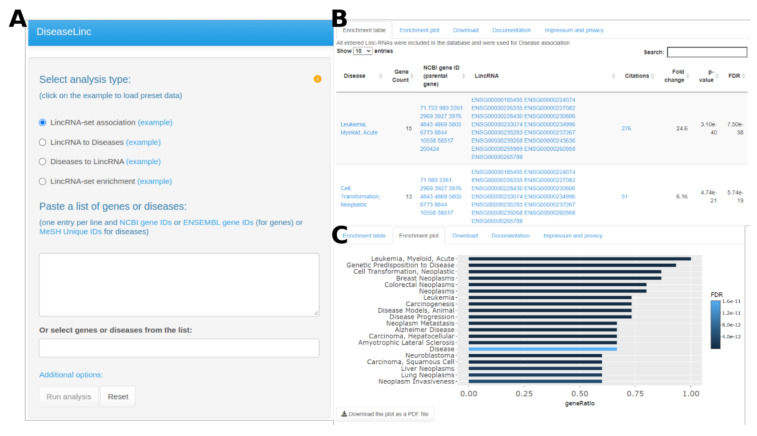
DiseaseLinc web interface. (**A**) The main analysis column displaying all possible analysis types, input fields, and parameters for fine-tuning the analysis; (**B**) an output table showing lincRNA and disease associations with statistical inference; and (**C**) a plot summarizing the top 20 diseases associated with an input set of lincRNAs.

The DiseaseLinc web server is based on manual annotations of biomedical literature data with MeSH terms. We extracted and used 2273 PubMed articles associated with at least one disease and one lincRNA parental gene (see the Materials and Methods section for details). The current version of the web server contains information of 172 lincRNAs related to 232 diseases. The tool is available at: http://cbdm-01.zdv.uni-mainz.de:3838/piyusmor/DiseaseLinc/, accessed on 31 December 2020. A user can perform four types of analysis, including LincRNA set association, LincRNA to Diseases, Diseases to LincRNA, or LincRNA-set enrichment (Figure 1A). The LincRNA-set association option considers a set of lincRNAs (two or more). These lincRNAs are considered altogether for their association with various diseases. LincRNA to Diseases and Diseases to LincRNA consider individual lincRNA and diseases, respectively, and report the corresponding associations for each individual entry that we obtained by analysis of the 2273 PubMed articles discussing lincRNAs and diseases. LincRNA-set enrichment accepts a set of lincRNAs (two or more) with gene-level statistics (e.g., expression level or fold changes between two biological conditions). A user can enter either NCBI gene IDs or ENSEMBL gene IDs for the lincRNAs and MeSH Unique IDs for the diseases. LincRNAs (ENSEMBL gene IDs) or diseases (MeSH headings) can also be selected from a drop-down list.

After submitting the analysis, the output is represented in seconds as a table and a plot, which can be downloaded for further exploration (Figure 1B,C). The output table also provides hyperlinks to corresponding Ensembl gene IDs, Entrez gene IDs, disease MeSH terms, and PubMed records. These linked records provide insight into the biomedical evidence supporting the associations reported.

### 3.2. LincRNA Expression Is Associated with Worse Prognosis in Breast Cancer

**Figure 2:**
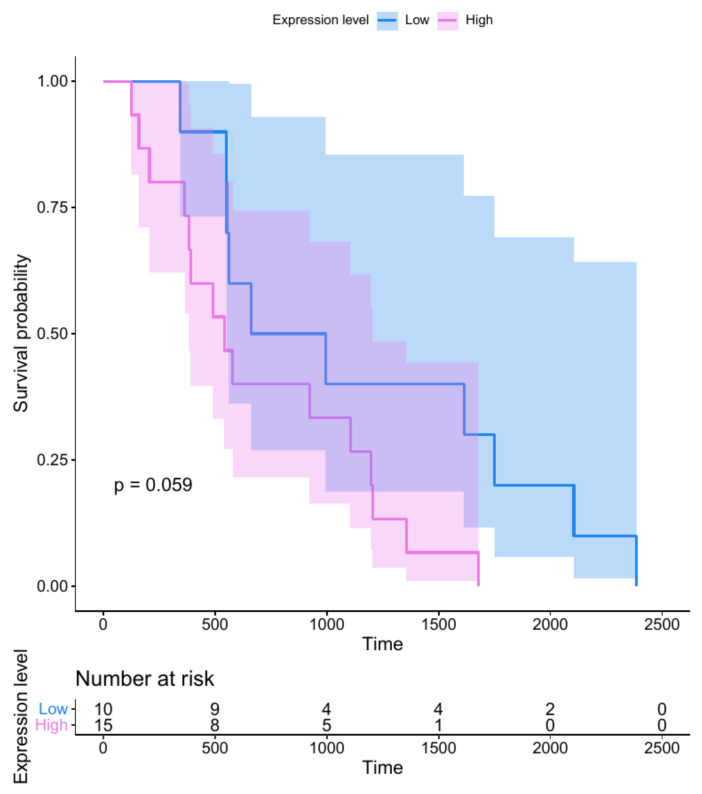
Kaplan–Meier curve comparing overall survival in breast cancer patients with high and low expression of the lincRNA ENSG00000260517, which has a potential effect on transcripts from *BANP* (a gene related to breast neoplasms).

We illustrated the potential of the associations underlying DiseaseLinc with one of the diseases often discussed in the literature corresponding to our set of selected parental genes of lincRNAs. Out of 172 lincRNAs from the database, parental genes of 72 lincRNAs were associated with breast neoplasms (Supplementary Table S1). We analyzed the expression of these lincRNAs in a panel of breast cancer patients (from the atlas of long non-coding RNA in cancer, TANRIC; [15]) (see the Materials and Methods section for details). Out of 72 lincRNAs associated with breast neoplasms, 45 lincRNAs were included in the dataset obtained from TANRIC. The correlation of expression level with the overall survival revealed that the expression of a number of these lincRNAs (ENSG00000224074, ENSG00000234718, ENSG00000234996, ENSG00000254973, ENSG00000255193, ENSG00000260517, and ENSG00000261480) was associated with poorer overall survival in breast cancer patients. Figure 2 shows the association for ENSG00000260517. DiseaseLinc mapped this lincRNA to the parental gene *BANP* (a.k.a. *SMAR1*), for which we recorded a significant number of publications (nine) discussing this gene in the context of breast neoplasms. Data for the other lincRNAs are shown in Supplementary Figure S1.

## 4. Discussion

Here, we presented DiseaseLinc, a web tool that provides enrichment analysis for datasets of lincRNAs. We used associations of lincRNAs to protein-coding genes that were obtained in a previous study, which employed a carefully designed sequence similarity method [11]. This allowed us to overcome the lack of functional annotations for lincRNAs, while focusing on a set with increased potential relevance as competitors of their associated genes. As the tool is based on the manual annotation of biomedical literature by diseases and genes, the database will expand with the growing literature. The tool will be updated yearly using an automated script.

Our results report enrichment in terms of human diseases selected by analysis of associations between genes and diseases linked in the PubMed database. The statistics reported are simple, and links to the PubMed records used as evidence allow the evaluation of each result, facilitating the bibliographic search for potential mechanisms that link lincRNAs, target genes, and disease.

Most of the lincRNAs from our dataset are associated with malignant diseases, including breast, colorectal, and prostatic neoplasms. Genetic instability is one of the major hallmarks of malignant cells [17]. Considering the diverse roles of lincRNAs in regulating gene expression, understanding their involvement in driving malignant transformation can provide an opportunity for therapeutic intervention [18,19]. Our illustrative example using a breast cancer dataset derived from the Cancer Genome Atlas demonstrates that our tool is helpful to filter from a list of differentially expressed lincRNAs the ones that can be more directly linked to a particular disease based on the available literature. The tool can either be used individually to filter disease-relevant lincRNAs or in tandem with specialized databases, like TANRIC [15], to support functional annotation with literature data. We expect that our tool will simplify the identification of disease-relevant lincRNAs and facilitate hypotheses concerning novel therapeutics.

## Data Availability

All data is available as supplementary material.

## Supplementary Materials

The following are available online at https://www.mdpi.com/article/10.3390/cells10040751/s1, Figure S1: Kaplan–Meier curves for the lincRNAs affecting the survival of breast cancer patients, Table S1: Diseases associated with 172 lincRNAs ranked by false discovery rate (FDR).
