# Supplementary material for: DiseaseLinc: Disease Enrichment Analysis of Sets of Differentially Expressed LincRNAs
Source: Cells. 2021 Mar 29;10(4):751. doi: 10.3390/cells10040751 (PMC8065951; doi:10.3390/cells10040751)
Supplement: Supplementary file 1 [file cells-10-00751-s001.zip › Supplementary_FigureS1.docx]

**Supplementary Figure S1**

**
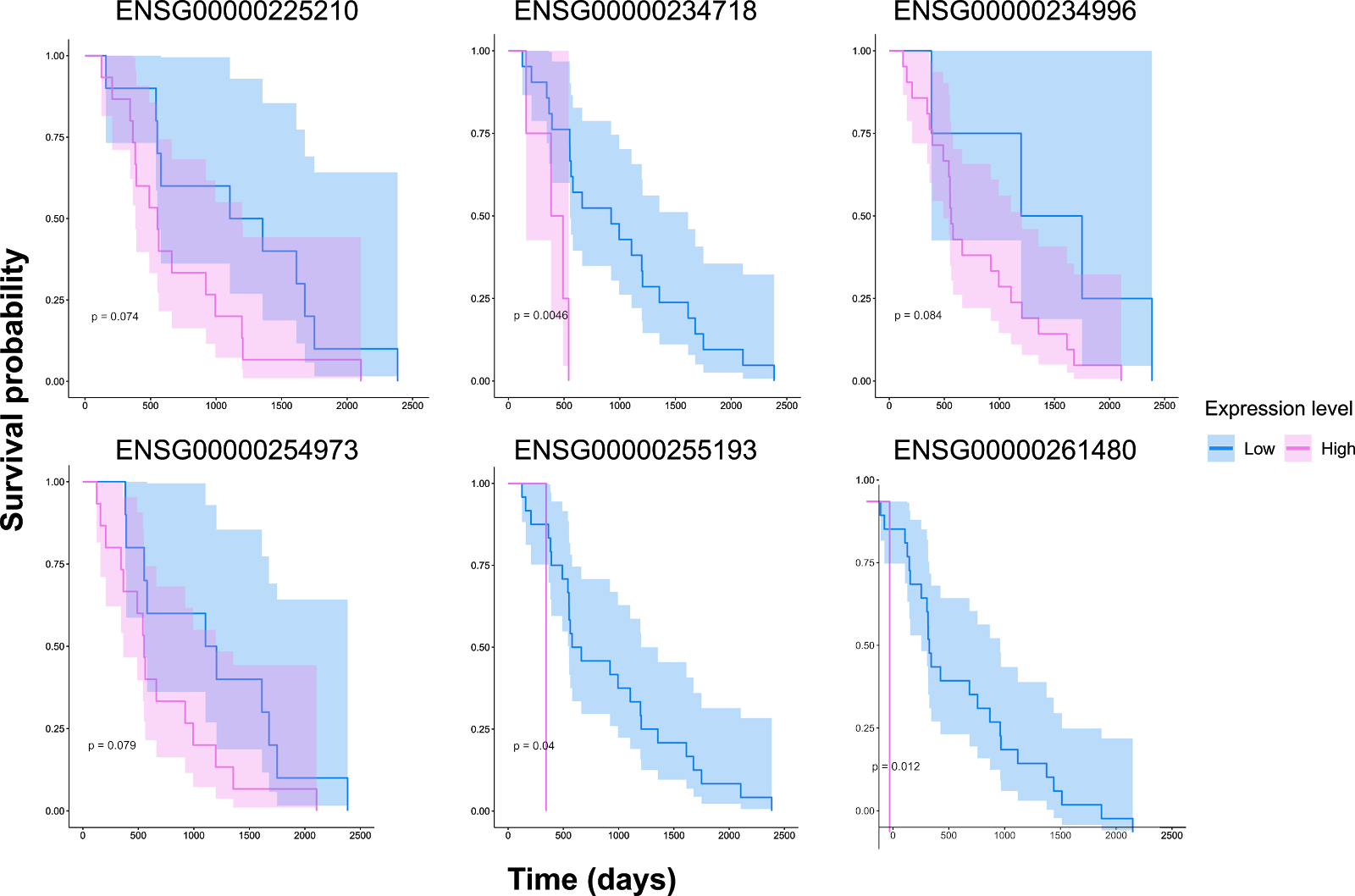
**

**Supplementary Figure 1.** Kaplan-Meier curves for the lincRNAs affecting the survival of breast cancer patients.
